# Epidemiology of campylobacteriosis in Germany – insights from 10 years of surveillance

**DOI:** 10.1186/1471-2334-14-30

**Published:** 2014-01-15

**Authors:** Anika Schielke, Bettina M Rosner, Klaus Stark

**Affiliations:** 1 Department of Infectious Disease Epidemiology, Gastrointestinal Infections, Zoonoses and Tropical Infections Unit, Robert Koch Institute (RKI), Seestraße 10, 13353 Berlin, Germany

**Keywords:** *Campylobacter*, Surveillance, Germany, Epidemiology

## Abstract

**Background:**

Campylobacteriosis caused by *Campylobacter* spp. is the most common notifiable bacterial gastrointestinal disease in Germany and a major problem in many other European countries as well. In contrast to other infectious diseases, e.g., salmonellosis, the annual number of notified campylobacteriosis cases has increased in Germany and other European countries from 2001–2010.

**Methods:**

National surveillance data from 2001 through 2010 were the basis of a detailed description of the epidemiological pattern of *Campylobacter* infections in Germany. Special focus was placed on geographical distribution and time trends of *Campylobacter* infections as well as the identification of risk groups.

**Results:**

In total, 588,308 cases of campylobacteriosis were recorded during the observed time period. The mean annual incidence increased from 67 cases/100,000 population in 2001 to 80/100,000 population in 2010. Almost 92% of the notified *Campylobacter* infections were acquired in Germany. A seasonal distribution was observed with a large peak in the summer months and a small peak in January. Incidence was highest in children ≤4 years and young adults 20–29 years of age. Especially young children living in rural regions in Germany seemed to be at high risk of *Campylobacter* infection.

**Conclusions:**

*Campylobacter* is the leading cause of bacterial gastroenteritis in Germany, and has been of rising public health concern. There is a need for enhanced prevention of *Campylobacter* infections and the data presented here may contribute to better target prevention measures with focus on identified risk groups such as children and young adults.

## Background

Campylobacteriosis is the most frequently reported zoonosis in the European Union (EU) and the most common notified bacterial gastrointestinal disease in Germany [1]. In contrast to other infectious diseases, e.g., salmonellosis, the annual number of notified campylobacteriosis cases has increased in many European countries in recent years [1]. In the United States, *Campylobacter* infection is the second most common cause of bacterial enteritis after salmonellosis*,* with 2.4 million cases estimated to occur per year [2,3]. Thus, the burden of gastrointestinal disease due to *Campylobacter* is substantial.

Several *Campylobacter* species are known to be pathogenic to humans [4], with *Campylobacter jejuni* being the leading cause of campylobacteriosis worldwide, followed by *C. coli*[5]. *Campylobacter* can be transmitted human-to-human by the faecal-oral route. However, zoonotic or foodborne transmission predominates and *Campylobacter* is held responsible for a considerable part of foodborne infections [6].

The bacteria are widespread in the environment and have been detected in various animal reservoirs, for example, poultry, cattle, swine, and dogs [5]. Prevalence of *Campylobacter* is particularly high in chickens; therefore, contact to chickens and consumption of chicken meat are regarded as important risk factors for campylobacteriosis [5,7,8]. Although *Campylobacter* requires special growth conditions and is not able to multiply in an aerobic atmosphere, the bacteria may survive on food or in the environment for several days [9,10]. Additionally, the infective dose for humans is very low [11].

The incubation period typically varies from one to seven days before diarrhoea, abdominal cramps and fever may occur as the most common symptoms [4]. The disease is self-limiting and symptoms typically disappear within one to three weeks. Reactive arthritis and the Guillain–Barré syndrome are rarely observed sequelae of a *Campylobacter* infection [12].

The aim of this report is to describe demographic and geographic determinants and trends of campylobacteriosis in Germany from 2001 through 2010 in order to identify possible starting points for appropriate countermeasures and to provide a basis for disease management decisions.

## Methods

According to the Protection against Infection Act that has been in effect in Germany since 2001, direct or indirect detection of enteropathogenic *Campylobacter* is notifiable, if indicating an acute infection in humans. Primary diagnostic laboratories are obligated to inform the responsible local health department in the district where the patient resides. Anonymised notification data are electronically forwarded by the local health department via the state health department to the Robert Koch Institute (RKI), the federal public health institute.

We analysed the national surveillance data on notified *Campylobacter* infections from 2001 through 2010. Only cases fulfilling the reference definition were included in data analysis, which comprises cases with at least one clinical symptom of campylobacteriosis (diarrhoea, abdominal pain, fever) and with either a laboratory confirmed *Campylobacter* infection or an epidemiological confirmation, which includes a link to a laboratory-confirmed case or to a contaminated food item.

Routine surveillance data include information on age, sex, district of place of residence, date of disease onset, country/district of exposure, hospitalisation (admittance to hospital) and the detected *Campylobacter* species. We counted only hospitalisations with a disease onset before the hospitalisation date so that solely hospitalisations due to a campylobacteriosis were included in the analysis. These data are available through the national level database (SurvNet) at the RKI. Data are publically accessible via SurvStat@RKI (http://www3.rki.de/SurvStat/). Population data provided by the Federal Statistical Office have been used for calculation of campylobacteriosis incidence. Urban and rural regions were classified according to the criteria proposed by the Federal Institute for Research on Building, Urban Affairs and Spatial Development (German: Bundesinstitut für Bau-, Stadt- und Raumforschung [BBSR]). Cities with more than 100,000 inhabitants and districts with more than 150 inhabitants per km^2^ were defined as urban. Accordingly, cities with less than 100,000 inhabitants and districts with less than 150 inhabitants per km^2^ were classified as rural [13]. A variable termed “geographic setting” was formed with the two categories urban and rural.

Data analysis was conducted with Microsoft Excel 2010, and geographic maps were prepared with Regiograph version 11 (GfK GeoMarketing GmbH, Bruchsal, Germany). Statistical calculations were performed with Stata 12 (Stata Corporation, College Station, TX; USA). Poisson regression analysis with offset equal to the population size was conducted to estimate incidence and associated 95% confidence intervals (CI) and for group comparisons. *P* values <0.05 were considered statistically significant.

## Results

### Demographic distribution

**Figure 1:**
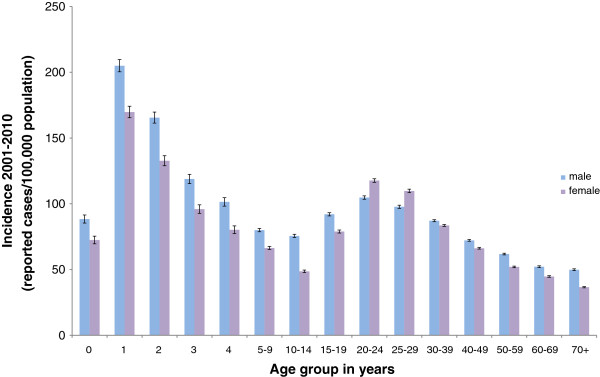
**Incidence of campylobacteriosis in Germany by age group and sex, 2001–2010.** Note that age group ranges vary.

From 2001 through 2010, a total of 588,308 cases fulfilling the reference definition were recorded in Germany. Most of these cases occurred sporadically (97%) with only a small proportion of cases reported in relation to outbreaks. The mean annual incidence was 72 cases/100,000 population ranging from 58/100,000 in 2003 to 81/100,000 in 2007. Mean incidence was slightly higher for men (96/100,000 population) than women (83/100,000 population) with an incidence rate ratio (IRR) of 1.15, *P* < 0.001. Incidence was highest in children 0–4 years of age compared to all other age groups (123/100,000 and 69/100,000 population, respectively, *P* < 0.001), particularly in one-year-old boys (205/100,000 population). A high incidence also occurred in young adults 20–29 years of age compared to all other age groups (107/100,000 and 67/100,000 population, respectively, *P* < 0.001). In contrast to other age groups, among the 20-29-year-olds, women were more frequently affected than men (113/100,000 and 101/100,000 population, respectively, *P* < 0.001) (see Figure 1). The majority of notified *Campylobacter* infections was acquired in Germany. Only 8% of cases were reported as travel-related. The most frequently named countries of infection were Spain, Turkey, France, Italy and India. The proportion of travel-related cases was highest in persons 25–39 years of age (11%) and lowest in persons ≥70 years of age (3%).

### Seasonal distribution

**Figure 2:**
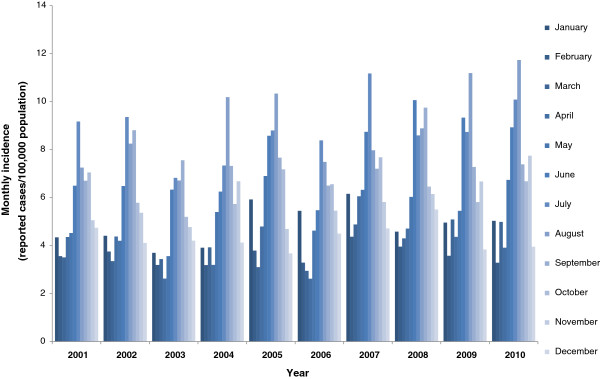
Seasonal distribution of reported campylobacteriosis in Germany, 2001–2010.

For *Campylobacter* infections a marked seasonality was observed. Incidence increased strongly from May to July and peaked in August with a monthly incidence of about 9/100,000 population (see Figure 2). The high incidence of infections in the summer months was independent of the year of notification and the age group and was observed for urban as well as rural regions. Interestingly, in January, a small peak in the monthly incidence of notified *Campylobacter* infections with disease onset in the first days of January was recognizable in all age groups in most years in urban as well as rural regions. The proportion of travel-related infections was highest in April (10%) and from August to October (range: 10-12%).

### Geographic distribution

**Figure 3:**
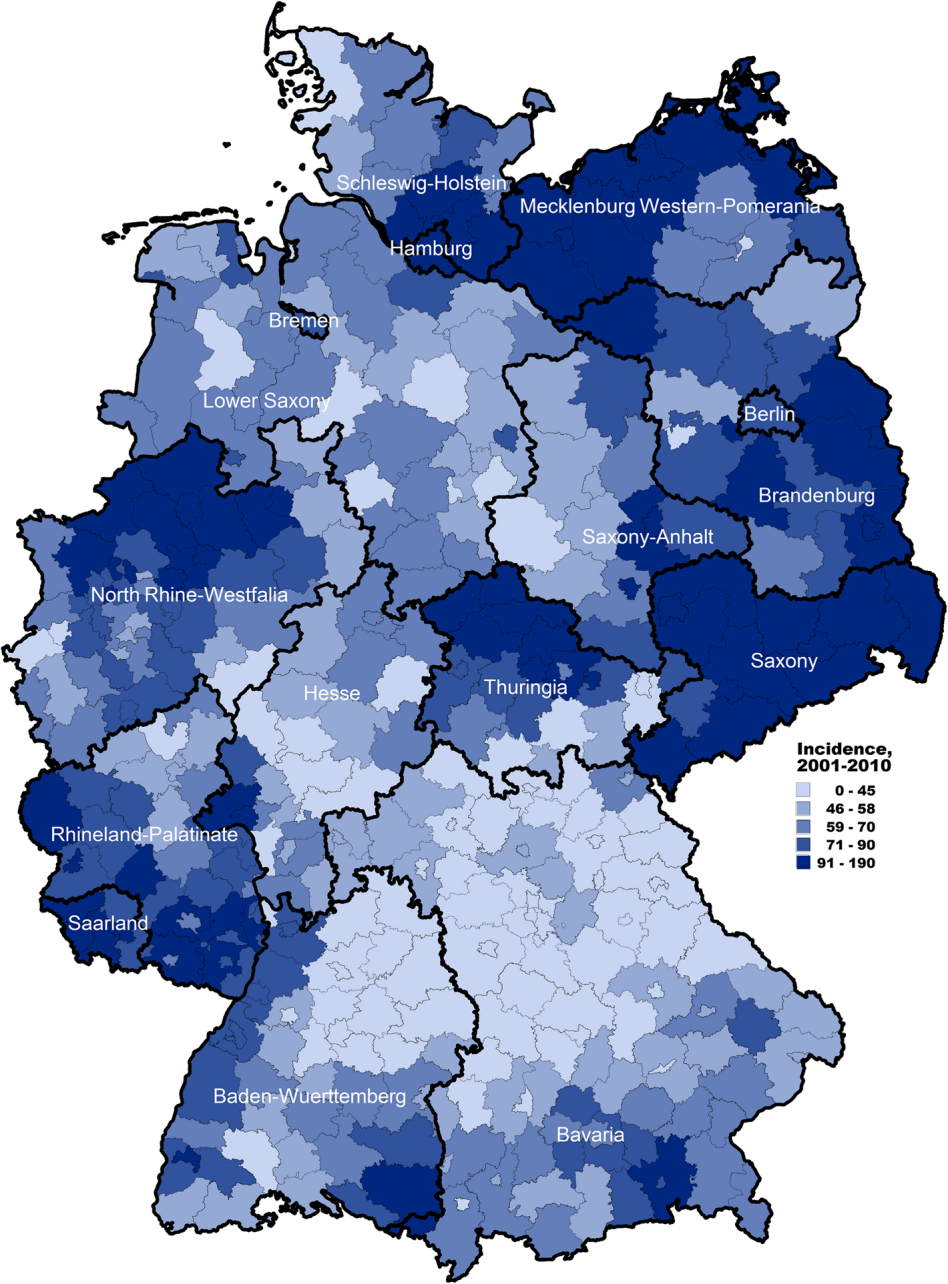
Geographic distribution of reported campylobacteriosis in Germany 2001–2010 (reported cases/100,000 population by district).

**Figure 4:**
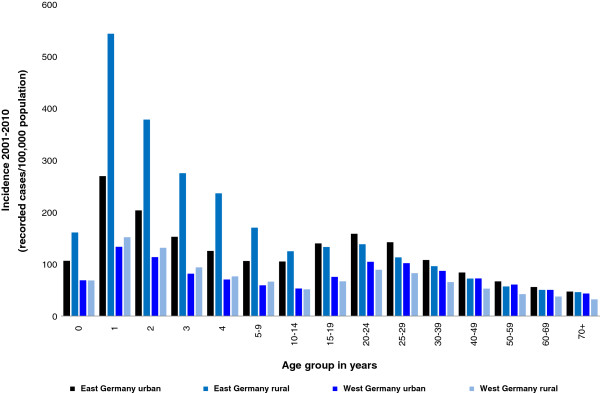
**Incidence of campylobacteriosis in Germany by age and geographic setting, 2001–2010.** Note that age group ranges vary.

The incidence of notified *Campylobacter* infections was higher in the eastern German federal states (Berlin, Brandenburg, Mecklenburg-Western Pomerania, Saxony, Saxony-Anhalt, and Thuringia) than in western German federal states (*P* < 0.001) (see Figure 3). In the eastern German federal states, the incidence in one-year-old children was more than threefold higher (414/100,000 population) compared with the incidence in this age group in western German federal states (138/100,000 population) (*P* < 0.001). Additionally, in eastern Germany, the incidence in one-year-old children was about threefold higher than the incidence in young adults 20–24 years of age, whereas in western Germany, the incidences in these two age groups were similar (see Figure 4).

### Urban–rural differences

The incidence in rural regions was 69/100,000 population and in urban areas 73/100,000 population (*P* < 0.001). Seasonality with high incidence in the summer months was observed in both settings. Children living in rural regions in Germany were more frequently affected by *Campylobacter* infections than children living in urban regions, which is especially obvious in the age group <10 years of age (130/100,000 and 83/100,000; *P* < 0.001). Vice versa, for higher age groups, incidences were higher in persons living in urban regions than in persons living in rural regions of Germany. The incidences in 20-69-years-old living in a rural or an urban setting were 64/100,000 and 77/100,000, respectively (*P* < 0.001) (see Figure 4).

### *Campylobacter* species and diagnostics

For 67% of the notifications a *Campylobacter* species was reported. Of those, 90% were caused by *C. jejuni*, about 7% by *C. coli,* and about 2% by other *Campylobacter* species, with *C. lari*, *C. fetus* and *C. upsaliensis* being the most frequently reported other species. For 33% of notified *Campylobacter* infections, differentiation between the species *C. jejuni* and *C. coli* had not been performed, or the species had not been typed at all, or was not reported. In rural as well as urban regions, *C. jejuni* was the main cause of campylobacteriosis. Increased incidence in the summer months was observed for both *C. jejuni* and *C. coli*, independent of the geographic setting. Cultivation is the “gold standard” for detection of *Campylobacter* and 86% of notified cases were diagnosed on the basis of this method. Enzyme immunoassays (EIA) were used for diagnosis in about 9% of notified cases. A combination of different detection methods was conducted in about 3% of notified cases, primarily cultivation in combination with EIA. In about 2% of notified cases the diagnostic method was not reported.

### Hospitalisation

On average, 10% of campylobacteriosis cases were reported as having been hospitalised because of the disease, which would correspond to a total number of 58,000 cases. Hospitalisation was more common among children <1 year of age (14% of cases) and elderly persons ≥70 years of age (22% of cases).

### Time trends

**Figure 5:**
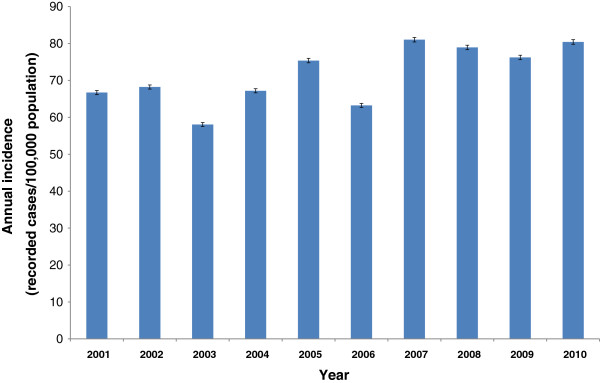
Annual incidence of campylobacteriosis in Germany, 2001–2010.

Overall, the annual incidence has increased significantly since 2001 (*P* < 0.001) resulting in more than 65,000 reported infections in 2010. This corresponds to an incidence of 80/100,000 population. Merely in 2003, 2006, and 2009 incidence was lower than in the respective previous year (see Figure 5). The strongest increase in the number of notified cases in the observed time period was recognized in the age group ≥15 years, especially in persons ≥50 years of age where the incidence increased from 39/100,000 population in 2001 to 61/100,000 population in 2010. In contrast, the incidence among children <10 years of age decreased in the same time period from 106/100,000 to 83/100,000. The proportion of hospitalisations increased from 9% in 2001 to 15% in 2010 (*P* < 0.001). The proportion of imported infections decreased from 11% in 2001 to 7% in 2010 (*P* < 0.001).

## Discussion

German surveillance data on campylobacteriosis were analysed for a ten-year period from 2001 through 2010. The incidence of *Campylobacter* infections in Germany was high with a mean annual incidence of 72/100,000 population. The overall increase over the observed time period was mainly due to a rise of domestically acquired cases.

The disease burden of bacterial enteritis due to *Campylobacter* is substantial in many European countries. In he EU, campylobacteriosis is the most commonly reported zoonosis with *C. jejuni* accounting for most of the confirmed cases [1,2]. An upward trend of the incidence of *Campylobacter* infections in the EU has been observed since 2005. The incidence in Germany exceeds the European average incidence of *Campylobacter* infections (80/100,000 vs. 49/100,000 in 2010). However, the incidence of campylobacteriosis varies widely among the reporting EU countries, most likely due to different surveillance and health care systems. Also, differences in *Campylobacter* related risk factors, e.g. diet patterns, contamination levels of different food items, or climatic factors, may play a role [14]. Sero-epidemiological studies indicate that under-ascertainment of *Campylobacter* infections is likely [15-17]. Analysis of routine surveillance data is mainly limited by under-ascertainment and under-reporting. Notified cases may not be representative of all *Campylobacter* infections in the population because diseased persons, especially those with mild infections, do not necessarily seek medical care and, in addition physicians do not always order stool analysis from patients with diarrheal diseases. Both are prerequisites for notification. Furthermore, diagnosed infections may not always be reported to the health authorities despite being mandatory by law.

Campylobacteriosis is assumed to be mainly a food-borne disease and the consumption of poultry is one major risk factor [5,7,8]. In contrast to the consumption of pork or beef, the annual consumption of poultry increased from 1996 to 2006 in Germany (from 8.4 to 9.9 kg per capita) [18]. This may partly explain the increase in the incidence of *Campylobacter* infections in Germany, as poultry is frequently contaminated with *Campylobacter*. Routine monitoring of zoonotic pathogens in food and farmed animals in Germany revealed that the prevalence of *Campylobacter* in poultry meat ranged from 14% to 34% per year. In contrast, contamination of beef and pork with *Campylobacter* was markedly lower [1,19]. The prevalence of *Campylobacter* in chickens ranged from 6% to 64% per year [1,19]. However, a distinct trend in the annual prevalence of *Campylobacter* in poulty meat and in chickens was not observed for the years 2001–2010. In contrast to *Campylobacter* infections, the number of human infections due to *Salmonella enterica* serovar Enteritidis has decreased in recent years, which is mainly attributed to successful *Salmonella* control programmes in poultry including immunisation [1,19]. To date, programmes to control *Campylobacter* in poultry have not been implemented successfully.

In Germany, incidence of campylobacteriosis was particularly high in the years 2002, 2005 and 2007. Several explanations are possible. The low number of *Campylobacter* cases reported in 2001 may reflect an artefact of reporting because the Protection against Infection Act was newly introduced that year and the surveillance system was still in its infancy. In 2002, the surveillance system was fully established resulting in a higher number of notified cases. In 2004, diagnostic criteria for the campylobacteriosis case definition for notifications were modified to include EIA and enzyme-linked immunosorbent assays (ELISA) [20]. Using these antigenic tests, non-culturable bacteria can be detected as well. Thus, the enhanced use of these test systems most likely resulted in the strong increase in notified cases in 2005. Various possible reasons have been discussed for the elevated case numbers in 2007 [20]. For instance, the prevalence of *Campylobacter* in routinely tested chickens and chicken herds was higher in 2007 and the prevalence of *Campylobacter* in poultry and chicken meat was increased in this year compared to previous years [21]. Furthermore, warm weather in the spring of 2007 may have triggered recreational activities with enhanced exposure to possible risk factors, for example consumption of undercooked meat at barbecues [8], or swimming in contaminated water bodies [7].

A correlation between temperature and number of campylobacteriosis cases has been described before and may also explain the seasonal pattern of the disease with an incidence peak in the summer months, which has been described for many countries [22,23]. In Germany, *Campylobacter* incidence peaks both in rural as well as urban areas in the summer. Such a seasonal pattern is also characteristic for other zoonotic enteric diseases of bacterial origin, e.g., salmonellosis*,* indicating similar transmission routes [1,22]. Contamination of broilers and chicken meat with *Campylobacter* tends to be higher in the summer months. The contamination rate of *Campylobacter* in broilers was found to be highest in August [24]. Chicken legs at retail examined during a one-year study in Germany showed two peaks of contamination, one from February to March and the second from July to August [25].

Interestingly, in addition to the incidence peak in the summer months, we observed a second, smaller incidence peak of campylobacteriosis in January. This peak was not due to a reporting delay over the Christmas and New Year’s holidays but was caused by an increase of infections with disease onset in the early days of January, which may indicate exposure of patients to *Campylobacter*-contaminated food items at the end of the previous year, possibly on New Year’s eve. Further studies would have to be conducted to elucidate this incidence increase.

In Germany as well as in the EU, incidence was highest among children under four years of age and, in particular, one-year-old boys were affected. This is in line with the epidemiology of other gastrointestinal infectious diseases showing a comparable demographic pattern, e.g., infections with *Yersinia enterocolitica*, *Salmonella* spp., and Shiga toxin-producing *Escherichia coli* (STEC) with peak incidence in young children [26-28]. The immune response in infants has not fully established, which may explain their susceptibility to various infectious diseases. However, other age-specific risk factors, like e.g. insufficient hand hygiene or close contact to animals or to the environment, may also play a role.

A high incidence of campylobacteriosis was also found in adults aged 20–29 years in Germany. Interestingly, women were more frequently affected than men in this age group in contrast to all other age groups, in which men were more frequently infected. This age and gender distribution has also been reported for other countries (England, Wales, and the Netherlands) [15,29]. Possible reasons may be a higher risk of exposure in women of this age group due to human-to-human transmission from young children they take care of or because they prepare and eat chicken more frequently than men of this age group, possibly resulting in *Campylobacter* infections if food items are not sufficiently heated or cross-contaminated.

The German surveillance data show that children living in rural regions were more frequently affected by *Campylobacter* infections than children living in urban areas. In contrast, this effect was not observed or even reversed in older persons (Figure 4). Our result is consistent with a study conducted in the German federal state of Hesse from 2005–2006 [30] that examined the association between age-specific *Campylobacter* incidence and the degree of urbanicity of the district of the place of residence also showing an increased incidence in children living in rural areas. This has also been demonstrated by other studies in Europe [23,31]. It is conceivable that children in rural areas may be more exposed to farm animals, including chickens, and to other possible sources of *Campylobacter* (e.g., contaminated water, wild birds) than children living in urban areas [8].

## Conclusions

Surveillance of notifiable diseases provides valuable data on demographic and geographic determinants as well as trends of campylobacteriosis in Germany. Children and young adults, and particularly children living in rural regions, were identified as groups at high risk of *Campylobacter* infection. Efforts should be undertaken to control campylobacteriosis and reduce infection risks particularly in these risk groups. Prevention measures should include strengthening efforts to reduce *Campylobacter* prevalence in farm animals and food as well as providing detailed information on the risks of *Campylobacter* infection to the target groups. We were able to identify vulnerable population groups in our analysis. However, the associated risk factors are not known in detail. Thus, the investigation of age-specific and season-related as well as geographic setting-related risk factors should be the focus of further research.

## Abbreviations

BBSR: Federal institute for research on building, urban affairs and spatial development (German: Bundesinstitut für Bau-, Stadt- und Raumforschung); CI: Confidence interval; EIA: Enzyme immunoassays; ELISA: Enzyme-linked immunosorbent assay; EU: European union; IRR: Incidence rate ratio; RKI: Robert Koch Institute; STEC: Shiga toxin-producing *Escherichia coli.*

## Competing interests

The authors declare that they have no competing interests.

## Authors’contributions

AS analysed the data and wrote the manuscript. BR and KS conceptualized the data analysis and contributed to the manuscript. All authors read and approved the final manuscript.

## Pre-publication history

The pre-publication history for this paper can be accessed here:

http://www.biomedcentral.com/1471-2334/14/30/prepub
